# Ovarian Abscess Following Therapeutic Insemination

**DOI:** 10.1155/S1064744994000189

**Published:** 1994

**Authors:** Bradford A. Kolb, Lane Mercer, Albert J. Peters, Ralph Kazer

**Affiliations:** Department of Obstetrics and Gynecology Northwestern University Medical School 333 East Superior Street #420 Chicago IL 60611-3095 USA

## Abstract

*Background:* Artificial insemination is a commonly performed procedure for the treatment of various forms of infertility. Infectious complications have only rarely been noted as a complication of intrauterine insemination (IUI).

*Case:* In this presentation, we report the first case of an ovarian abscess following IUI with the husband's semen. Despite treatment with triple antibiotics, an oophorectomy was required. Surgical as well as pathological evaluation confirmed the diagnosis of an ovarian abscess. Following surgery, the patient responded well to antibiotic therapy.

*Conclusion:* Since pelvic infections are ascending processes, the violation of the natural cervical barrier with IUI can theoretically place the patient at increased risk for this complication. While few advocate routine cultures of semen samples, the clinician must be acutely alert to potential infectious morbidity following this procedure. Early diagnosis and intervention are necessary to minimize morbidity and optimize treatment.


Infectious Diseases in Obstetrics and Gynecology 1:249–251 (1994)
© 1994 Wiley-Liss, Inc.

### Ovarian Abscess Following Therapeutic Insemination

**Bradford A. Kolb, Lane Mercer, Albert J. Peters, and Ralph Kazer**
*Department of Obstetrics and Gynecology, Northwestern University Medical School, Chicago, IL*

Abstract

***Background:*** **Artificial insemination is a commonly performed procedure for the treatment of various forms of infertility. Infectious complications have only rarely been noted as a complication of intrauterine insemination (IUI).**

***Case:*** **In this presentation, we report the first case of an ovarian abscess following IUI with the husband's semen. Despite treatment with triple antibiotics, an oophorectomy was required. Surgical as well as pathological evaluation confirmed the diagnosis of an ovarian abscess. Following surgery, the patient responded well to antibiotic therapy.**

***Conclusion:*** **Since pelvic infections are ascending processes, the violation of the natural cervical barrier with IUI can theoretically place the patient at increased risk for this complication. While few advocate routine cultures of semen samples, the clinician must be acutely alert to potential infectious morbidity following this procedure. Early diagnosis and intervention are necessary to minimize morbidity and optimize treatment.** © 1994 Wiley-Liss, Inc.

Key words
Infertility, infection, ovary, intrauterine insemination

Artificial insemination is a commonly performed procedure for the treatment of various forms of infertility. It can be defined as the mechanical deposition of semen in the vagina, cervical canal, or uterine cavity. Although rare, infectious morbidity can be associated with this procedure. The transmission of the human immunodeficiency virus (HIV) by artificial insemination with donor semen has been reported.[1,2] Other studies have indicated donor semen samples can contain ureaplasm, *Chlamydia trachomatis*, and hepatitis B.[2] In spite of these reports, pelvic inflammatory disease has only rarely been noted as a complication of artificial insemination.[3] Recently, Sable et al.[4] reported the first case of a ruptured tubo-ovarian abscess (TOA) following intrauterine insemination (IUI). Since pelvic infections are ascending infections, the violation of the natural cervical barrier with IUI can theoretically place the patient at increased risk for infectious morbidity. A case of an ovarian abscess following IUI with the husband's sperm is reported and the possible pathophysiological mechanism of this is discussed.

### CASE REPORT

A 35-year-old female, $G_1P_{0010}$ with the diagnosis of stage-1 endometriosis presented for reproductive assistance due to infertility. After a 2-year attempt to conceive with timed intercourse, the patient underwent IUI using her husband's sperm following empiric controlled ovulation hyperstimulation with clomiphene citrate. The patient's gynecologic history was not remarkable except for stage-1 endometriosis diagnosed by laparoscopy 2 years prior to seeking reproductive assistance. The fresh semen sample was washed in a Tyrode culture medium containing 0.1% bovine serum albumin, centrifuged, resuspended, and filtered over wet glass wool. No preinsemination cultures were performed. The freshly prepared sample was deposited intra-

**Address correspondence/reprint requests to Dr. Lane Mercer, Department of Obstetrics and Gynecology, Northwestern University Medical School, 333 East Superior Street, #420, Chicago, IL 60611-3095.**

Received July 27, 1993
Accepted January 8, 1994

uterinely via a small catheter after washing the cervix with sterile Tyrode's solution. Five days later, the patient presented with complaints of a mild vaginal discharge and lower abdominal discomfort, greater on the left side. Physical examination revealed uterine and left adnexal tenderness; however, her temperature was not elevated. Furthermore, pelvic sonography failed to reveal any significant findings. She was subsequently given doxycycline, 100 mg twice a day, for a possible pelvic infection, although the source of her symptoms was believed to be mostly likely due to a ruptured ovarian cyst. The patient reported having some initial relief with antibiotic therapy. Six days later, the patient complained of increased abdominal pain and fever of 39.6°C. Physical examination revealed a tender 4 × 4-cm left adnexal mass and a white blood cell (WBC) count of $11.0 \times 10^3/\mu l$. Transvaginal sonography demonstrated a 4.5 × 4.4-cm left adnexal mass with uniform homogeneous low-grade internal echoes within the ovary. A urine pregnancy test was negative.

The patient was hospitalized with the diagnosis of pelvic inflammatory disease and treated with cefotetan, 2 g IV q 12 h. Due to the patient's persistent fever, antibiotic therapy was changed on hospital day 3 to ampicillin (2 g IV q 6 h), gentamicin (100 mg IV q 8 h), and clindamycin (900 mg IV q 8 h). Blood and cervical cultures were without growth at 72 h.

A repeat ultrasound on hospital day 7 revealed the cystic left adnexal mass to have increased to 4.5 × 6.0 cm, and to be bilobed, hyperechoeic, and homogeneous. A presumptive diagnosis of a pelvic abscess was made, and laparotomy was performed on the following day. Percutaneous catheter drainage was considered but due to its experimental nature and high rate of complications as well as patient desire for definative treatment, surgical drainage was performed. Dense pelvic adhesions encasing the left adnexa and obliterating the cul-de-sac were noted. The ovary and distal portion of the fallopian tube were friable with a brownish-green appearance, although the endosalpinx was only minimally involved. Upon manipulation of the ovary a thick yellow-white exudate was expressed. Since the overlying tube appeared necrotic and friable, a salpingo-oophorectomy was performed. Gross and microscopic evaluation of the ovarian tissues revealed marked edema and hemorrhage, while the distal portion of the fallopian tube which was adherent to the ovary displayed extensive perisalpingitis. Fluid from the abscess was collected by aspiration into a syringe and transported to the laboratory. Gram stain from the abscess cavity revealed the presence of neutrophils; however, no bacteria were present. All cultures were plated within 2 h of sampling. The specimen was plated onto 5% sheep blood agar, Columbia CNA agar, Mac Conkey agar, chocolate agar, and modified Thayer-Martin agar and into a thioglycollate broth for the recovery of aerobic organisms. For anaerobic isolates, the specimen was plated onto laked horse blood agar and 5% laked sheep blood with 50 μg/ml gentamicin and into enriched thioglycollate broth. All intraoperative cultures of the peritoneum and abscess cavity, both aerobic and anaerobic, were negative for growth. Postoperatively, the patient's condition rapidly improved while on antibiotic therapy. She was discharged home on postoperative day 4 with no further antibiotics.

Urethral cultures of the patient's husband were negative for *Neisseria gonorrhoeae* or *C. trachomatis*.

### DISCUSSION

Pelvic inflammatory disease is an ascending infection from the cervix, subsequently affecting the fallopian tubes and, if untreated, the ovaries. Although inciting organisms have been well identified, the bacteria most commonly isolated from pelvic infections are mixed aerobic and anaerobic species consistent with normal vaginal flora. It is believed that the cervix with its mucous barrier may protect against the ascent of potentially pathogenic bacteria.[5]

The patient reported herein underwent therapeutic IUI with her husband's sperm, thereby bypassing the normal physiologic barriers to infection of the upper genital tract. Based on the failure to find pathogenic organisms in the urethra of her partner, it is more likely that contamination with vaginal flora rather than direct infection by the husband's semen resulted in this episode of pelvic infection. While the patient was empirically started on doxycycline when she first presented with abdominal tenderness, a broader spectrum of coverage reflecting the natural vaginal flora may have helped to minimize infectious morbidity.

Ovarian abscess is a rare complication of pelvic infection and differs from the much more common TOA in that the ovarian stroma is the primary site of infection. While peritubal inflammation may be present, the endosalpinx is only minimally involved. A TOA, by contrast, involves the ovary by secondary spread from the infected tube. Most commonly, an ovarian abscess occurs after rupture of a follicular ovarian cyst with subsequent seeding of bacteria into the stigmata or colonization of the ovarian stroma.[6] The onset of an ovarian abscess is insidious and usually presents as pain and low-grade fever with acute peritonitis occurring from 1 week to 6 months following initial contamination. Unfortunately, there are few findings other than operative diagnosis that help in the differentiation between an ovarian abscess and a TOA. This patient, having undergone controlled ovarian hyperstimulation with clomiphene citrate in conjunction with timed insemination, may have been at increased risk for an ovarian infection due to the more extensive serosal disruption associated with this approach.

In any patient undergoing a transcervical procedure at the time of ovulation in whom nondescript symptoms of pelvic infection occur, an ovarian abscess should be considered. The treatment of an ovarian abscess, once suspected, is surgical intervention. In any patient undergoing IUI who has risk factors for sexually transmitted disease or a history of the same, antibiotic prophylaxis might be instituted. However, the side effects must be considered and antibiotics are not universally recommended. Generally, a history of salpingitis should be viewed as a contraindication to IUI.

Appropriate diagnostic modalities include evidence of systemic infection such as the sedimentation rate of C-reactive protein and ultrasound or computerized tomography for the presence of a fluid-filled cavity within the ovary. As with many abscesses, there may be little evidence of acute elevation of WBC count until rupture or overwhelming sepsis occurs. As antibiotic therapy is almost always unsuccessful, surgical intervention is the hallmark of therapy.[3] Oophorectomy with preservation of other reproductive structures can be accomplished if the diagnosis is made early and accurately.

Ovarian abscess following IUI is a rare complication. The combination of a transcervical procedure and controlled ovarian hyperstimulation may render this complication more likely. The clinician must be alert to the potential infectious morbidity following this procedure and the importance of early diagnosis and intervention in minimizing morbidity.

### REFERENCES

1. Hummel WP, Talbert LM: Current management of a donor insemination program. Fertil Steril 51:919–930, 1989.
2. Leiva JL, Peterson EM, Wetkowski M, de la Maza LM, Stone SC: Microorganisms in semen used for artificial insemination. Obstet Gynecol 65:669–672, 1985.
3. Chong AP, Taymor ML: Sixteen year's experience with therapeutic donor insemination. Fertil Steril 26:791–795, 1975.
4. Sable DB, Yanushpolsky EH, Fox JH: Ruptured pelvic abscess after intrauterine insemination: A case report. Fertil Steril 59:679–680, 1993.
5. Sparks RA, Purrier BG, Watt PJ, Elstein M: Bacteriological colonization of uterine cavity: Role of tailed intrauterine contraceptive device. Br Med J 282:1189, 1981.
6. Wetchler SJ, Dunn LJ: Ovarian abscess. Report of a case and review of the literature. Obstet Gynecol Surv 40:476–485, 1985.
